# Dystocia in Pet Giant African Land Snails

**DOI:** 10.3390/ani16162535

**Published:** 2026-08-14

**Authors:** Silvana Schmidt-Ukaj, Michaela Gumpenberger

**Affiliations:** 1 Clinical Unit of Internal Medicine Small Animals, Avian and Reptile Service, University of Veterinary Medicine Vienna, 1210 Vienna, Austria; 2 Clinical Unit of Diagnostic Imaging, University of Veterinary Medicine Vienna, 1210 Vienna, Austria; michaela.gumpenberger@vetmeduni.ac.at

**Keywords:** land snail disease, egg-binding, invertebrate medicine, gastropod mullusks, diagnostic imaging

## Abstract

Background: Giant African Land Snails originate from Africa. Although these snails are becoming more popular as exotic pets in Europe, there is a lack of comprehensive information about their diseases and how to treat them. Reported medical issues included failures in husbandry and feeding (often leading to aestivation, calcium deficiency or renal disease), trauma, poison, prolapse, and ecto- and endoparasites, as well as bacterial and fungal infections. Although their reproductive system has been well studied, dystocia has not yet been reported in terrestrial snail species. Patient Presentation: Two cases of dystocia in pet Giant African Land Snails are presented. Case 1 involved a 5.5-year-old *Lissachatina fulica* with esophageal prolapse and severe chronic dystocia, as indicated by radiographic and computed tomographic findings, ultimately leading to euthanasia. Case 2 involved a 3-year-old *Lissachatina albopicta* also affected by dystocia, with computed tomography again showing findings consistent with the diagnosis, but subsequently laying most of the pathological clutch with supportive, conservative treatment. Conclusion: Dystocia in Giant African Land Snails is a significant health concern that can lead to severe complications and mortality. Early diagnosis using imaging techniques is crucial for identifying reproductive issues and determining appropriate treatment strategies. However, because these terrestrial snails have an open circulatory system and vascular anatomy remains incompletely characterized, the reproductive tract remains difficult to access surgically. Furthermore, there is currently insufficient evidence to support hormonal induction of oviposition as a therapeutic approach for egg retention in this species. Consequently, conservative management remains the primary supportive therapy.

## Simple Summary

Giant African Land Snails are kept as exotic pets in Europe, and when they become ill, owners seek care from veterinarians. Unfortunately, the available veterinary literature on their diseases, diagnostic approaches, and treatment options is still limited and insufficient to serve as a reference. We present two snails with dystocia (egg binding), demonstrate how diagnostic imaging (radiography and computed tomography) helped identify the condition and discuss potential treatment approaches and their limitations. Given the current lack of knowledge regarding surgical and hormonal interventions in land snails, conservative management remains the most feasible approach for treating dystocia at present. These findings provide useful information for pet snail owners and veterinarians alike, contributing to better care for these increasingly popular pets.

## 1. Introduction

Giant African Land Snails (GALs), such as *Lissachatina* (*L.*) *fulica* and *L. albopicta*, originate from Africa. Although these snails are becoming more popular as exotic pets in Europe, there is a lack of comprehensive information about their diseases and how to treat them. During veterinary clinical examination, a systematic approach similar to that used in other animal species should be followed, beginning with a thorough husbandry and dietary history. An overview about basic anatomy, husbandry, handling, diseases, anesthesia and euthanasia in land snails is given by [1,2]. Reported medical issues included failures in husbandry and feeding (often leading to aestivation or calcium deficiency), trauma, poison, prolapse, and ecto- and endoparasites, as well as bacterial and fungal infections [1,2]. Renal diseases in GALs were previously described by [3]. To the author’s knowledge, there are no published studies or case reports describing dystocia in land snail species.

However, their reproductive system has been well studied. Terrestrial gastropods are hermaphrodites, possessing both male and female reproductive organs, as previously described by [4,5,6,7]. The genital system comprises, from posterior to anterior, the ovotestis, ovotestis duct, and spermoviduct with prostatic acini. It also includes the uterus, with associated calciferous glands and a sulcus. The vas deferens continues into the penis, while the oviduct leads to the vagina; both ultimately open into a common genital atrium located in the right head–neck region [5]. After reaching sexual maturity, typically between 6 and 8 months of age, the snails lay several egg clusters annually, each consisting of approximately 130–243 eggs and buried in substrate. Single eggs are white to lemon-yellow, calcareous, and measure approximately 4 mm × 5 mm [4,8,9,10,11].

Although these snails are increasingly kept as exotic pets in Europe, comprehensive information regarding their veterinary care remains scarce. To address this gap, we initiated an imaging study using computed tomography (CT), ultrasound, and radiography to visualize internal organ structures in GALs and provided reference data for diagnosing disease. Regarding the genital tract, calcified eggs are clearly visible on radiographs. The penis and the albumen gland can be distinguished using CT. The cranial portion of the penis, located on the right side of the snail’s head, can be differentiated sonographically as well. However, other parts of the reproductive system could not be identified [12]. The present paper aims to present two cases of dystocia in pet GALs, highlighting the importance of early diagnosis and management, and discuss potential treatment approaches and their limitations.

## 2. Case Reports

### 2.1. Case 1

A 5.5-year-old Giant African Land Snail (*L. fulica*), weighing 511 g, was presented to the emergency clinic with an esophageal prolapse, which was successfully relocated.

The snail was housed with up to five other *L. fulica* in a 130 cm long × 80 cm high × 75 cm deep aquarium maintained at >75% humidity and temperature ranging from 22.6 to 25 °C. An approximately 20 cm deep garden soil mixture, supplemented with lime, was provided as substrate. Its diet consisted of vegetables, animal protein and cuttlebone. The owner reported that the snail had not been observed laying eggs for the previous two years, whereas prior to this, it had regularly laid approximately 100 eggs once a month.

**Figure 1:**
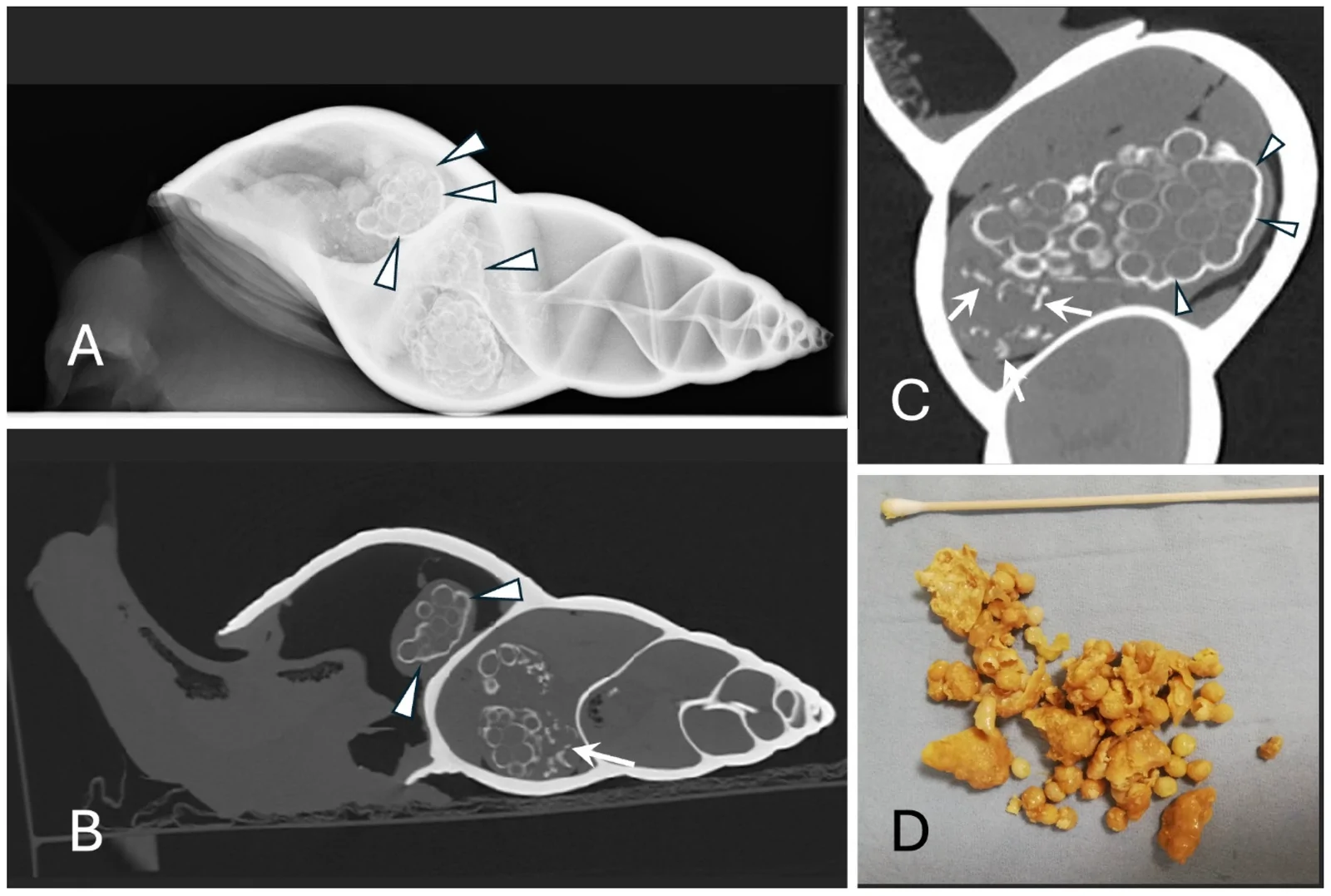
Computed Tomography (CT) (**A**) Lateral radiograph and CT images in the sagittal (**B**) and coronal (**C**) planes, displayed using a modified bone window of a Giant African Land Snail (Case 1). Multiple, calcified eggshells of various density and thickness are visible. An outer hyperdense layer encircles parts of the clutch (arrowheads). Multiple eggs are fractured (arrows). The heterogeneous appearance and the fractured shells indicate dystocia. Note that CT provided substantially greater anatomical detail, whereas radiography allowed only an approximate estimate of the number of eggs. The abnormal outer capsule (arrowheads) can be seen roughly. (**D**) Clinical picture of abnormal clutch, photographed after euthanasia for scientific documentation: yellowish-brown eggs that were partially fractured or fused; some eggs were arranged in layers and embedded within a hardened capsule.

In the following days, the snail underwent diagnostic imaging to investigate the underlying cause of the esophageal prolapse, as previously described by [12]. Plain radiographs were performed in dorsoventral and laterolateral views using 60 kV and 2.5 mAs, a film-focus distance of 50 cm and a digital mammography cassette system (Kodak DirectView PQ storage phosphor screen/cassette, Carestream, Health Inc., Rochester, NY, USA). For CT examination, the snail was placed in a plastic box and positioned perpendicular to the gantry. Sagittal scans were conducted with a 16-slice helical CT (Siemens Somatom Emotion, Vienna, Austria), using 80 mAs, 130 kV, rotation time 1.5 s, pitch 0.8, slice thickness 0.6 mm and a matrix of 512 × 512. Images were then reconstructed with soft-tissue and sharp bony kernel and evaluation was performed using modified soft-tissue and bone window settings. The images revealed over 100 eggs with variably shaped dense shells and a hyperdense outer rim surrounding the clutch. Multiple eggs were broken. CT provided greater anatomical detail than radiography. The imaging findings were consistent with severe chronic dystocia. Moreover, the hyperdense rim may have indicated adhesions or fibrosis (Figure 1).

Because the esophageal protrusion recurred and surgical intervention was considered prohibitively risky (the genital tract cannot be accessed surgically without significant blood loss), euthanasia was elected to prevent further suffering. Euthanasia was performed under general anesthesia with ketamine (100 mg/kg IM, Ketamidor^®^ 100 mg/mL, VetViva Richter GmbH, Wels, Austria) and xylazine (10 mg/kg IM, Sedaxylan^®^ 20 mg/mL, Eurovet Animal Health B.V., Bladel, The Netherlands), followed by pentobarbital (450 mg/kg IM, Release^®^ 300 mg/mL, VetViva Richter GmbH, Wels, Austria). Cardiac arrest was confirmed using Doppler ultrasonography. After death, the pathological clutch was photographed for scientific documentation and consisted of yellowish-brown eggs that were partially fractured or fused; some of them were arranged in layers and embedded within a hardened capsule (Figure 1D). However, neither a systematic necropsy nor histopathology was performed.

### 2.2. Case 2

A 3-year-old Giant African Land Snail (*L. albopicta*), weighing 259 g, was presented to the clinic because of apathy. The owner reported reduced activity, slowed locomotion, and decreased food intake since taking over the snail several months earlier. The snail was now housed with five other giant land snails, including *A. achatina* and *L. albopicta*. The terrarium had dimensions of 50 cm in length by 60 cm in height and 45 cm in depth. The temperature inside the terrarium was maintained at 25 °C, with a humidity level of 85–90% (using moss and manual spraying) and lime-amended coconut fiber was provided as substrate. The snails were fed a diet consisting of lettuce, zucchini, and cucumber. They also received cuttlebone as a calcium source, calcium powder, and crushed eggshells. Previous husbandry conditions and information regarding prior egg laying were unknown.

**Figure 2:**
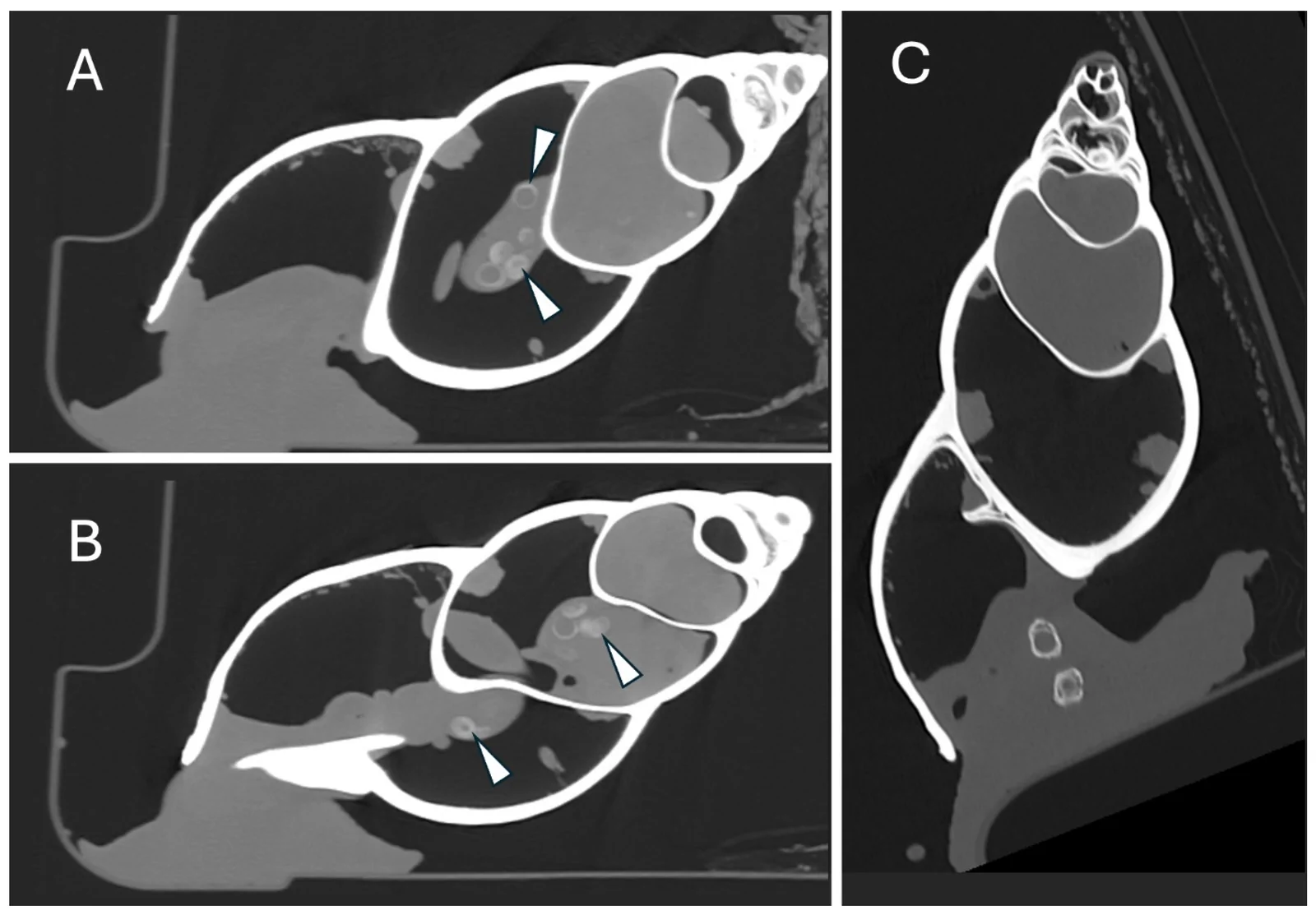
Computed Tomography (CT) images in a modified bony window in the sagittal and paramedian planes of a Giant African Land Snail (Case 2). Panels (**A**,**B**) represent the initial examination, in which 15 mineralized eggshells were observed (arrowheads); some were fractured, whereas others were irregularly thickened. Panel (**C**) is the follow-up examination one year later and shows two remaining, abnormally layered, thickened eggshells positioned at the level of the shell opening. Final diagnosis was chronic dystocia. In contrast to Case 1 (Figure 1), no hyperdense outer layer was observed around the clutch.

CT examination was performed as described in Case 1 for further evaluation. It revealed remnants of an old clutch with 15 broken and irregularly shelled eggs (Figure 2A,B).

The owner was instructed to maintain the snail under optimal husbandry conditions, including appropriate temperature and humidity levels and a substrate depth of 15–20 cm. Warm-water baths administered daily to several times per week and vegetable porridge with calcium powder were provided as additional supportive care. After 4 months the owner reported the laying of the pathological clutch. According to the owner, the eggs were broken, yellowish in color, irregularly shelled, and partially fused. On a follow-up CT examination one year later, only two eggs were present. They showed an irregular and thickened shell (Figure 2C).

## 3. Discussion

Currently, to the authors’ knowledge, no published studies or case reports have described dystocia in land snail species and no standardized definition of dystocia in gastropods has yet been established. In the cases presented here, the presence of heterogeneous, fragmented eggs, in one case packed in hyperdense layers, in contrast to the normally individually deposited, smooth-shelled eggs within a clutch, associated with clinical signs of apathy and esophageal prolapse, indicated findings consistent with pathological egg retention and dystocia.

Additionally, any discussion of possible causes remains speculative. In other exotic species, dystocia is a well-recognized multifactorial condition associated with systemic factors such as endocrine and metabolic disorders, hypocalcemia, infections, or mechanical obstruction, as well as environmental factors including inadequate temperature, humidity, or nesting conditions [13].

Ref. [14] reported that Giant African Land Snails actively explore their environment in search of suitable nesting sites with adequate moisture and substrate for oviposition. Ref. [8] investigated the reproductive behavior of *L. fulica* under experimental conditions, with particular emphasis on nest construction, nest depth, and clutch size. All egg clutches were deposited in self-constructed, 4.0 to 6.8 cm deep nests, indicating that nest construction is an important component of the oviposition behavior of this species. These findings indicate that sufficient and adequate substrate may therefore be important to facilitate normal oviposition behavior. In addition, previous studies have demonstrated that low dietary calcium levels combined with high stocking density can significantly reduce both laying rate and clutch size [15]. Unfortunately, previous husbandry conditions in Case 2 were unknown, but the owner of Case 1 provided appropriate substrate, humidity and calcium, whereas UVB lighting was not provided. Although it is difficult to determine whether calcium intake was adequate, consumption was monitored, with ingestion inferred from the presence of white fecal deposits. The absence of UVB exposure may have adversely affected calcium metabolism and, consequently, reproductive function, while the high stocking density in both cases may have further contributed to impaired reproductive performance.

Reproductive disorders are relatively common in other exotic animal species [16,17] and also have been occasionally seen in invertebrates [9]. These conditions can be further evaluated using diagnostic imaging techniques [18]. In the present land snail cases, both radiography and CT contributed to the identification of the underlying condition, while CT provided greater anatomical detail.

Therapeutic options for dystocia, including supportive care, medical management, and surgical intervention, depend on the type, chronicity and severity of the condition. Fragmented, heterogeneous eggs often necessitate surgical intervention in other species. However, due to the open circulatory system and the limited knowledge of vascular anatomy in GALs, the reproductive tract currently remains inaccessible for surgical approaches.

While the vascular anatomy of related species such as *Helix pomatia* has been described [19,20], no comparable data are available for GALs. In gastropods, hemolymph is not fully confined within vessels; instead, it circulates through a combination of arteries, veins, sinuses, and tissue spaces, directly bathing internal organs. Body cavities are separated by thin connective tissue membranes with small perforations. Furthermore, the hemolymph of GALs is colorless to slightly bluish, which complicates intraoperative visualization.

Although neuropeptides involved in the regulation of reproductive tract contractions and egg-laying behavior have been identified in gastropods, including fulicin in *L. fulica*, there is currently insufficient evidence to support hormonal induction of oviposition as a therapeutic approach for egg retention in this species [21].

Given the limited knowledge regarding surgical and hormonal interventions, conservative management remains the most feasible approach for treating dystocia in snails at present. In the first case, the severe chronic dystocia in combination with recurrent esophageal prolapse was considered indicative of an end-stage condition, and euthanasia was therefore elected to prevent suffering. In the second snail, dystocia was less advanced, allowing passage of most of the retained clutch during supportive conservative treatment, although two abnormal eggs remained. Notably, in one of the cases described here, dystocia was identified as a possible contributing factor to esophageal prolapse, whereas previous reports implicated renal enlargement as an underlying factor [3].

These findings highlight the need to expand current knowledge of disease processes and therapeutic options in GALs. Veterinarians should be aware of dystocia as a potential condition in these animals and consider advanced imaging modalities such as radiography or even CT to enable early and accurate diagnosis.

## 4. Conclusions

Dystocia represents a significant health concern in pet GALs, with the potential to cause severe complications or even mortality. Early diagnosis using a systematic approach similar to that used in other animal species is essential for improving outcomes and should begin with a thorough husbandry and dietary history, clinical examination and further diagnostic evaluation, such as advanced imaging techniques. Further research is urgently needed to enhance our understanding of GAL physiology and pathology and to develop effective diagnostic and therapeutic strategies tailored to this unique species.

## Data Availability

The original contributions presented in this study are included in the article. Further inquiries can be directed to the corresponding author.
